# Global C3 lowering in adulthood protects against hippocampal dysfunction and cognitive impairment in aged mice

**DOI:** 10.21203/rs.3.rs-6924607/v1

**Published:** 2025-06-23

**Authors:** Andre F. Batista, Jessey Presumey, Brijendra Singh, Maren K. Schroeder, Khyrul Khan, Emma Spooner1, Shaomin Li, Michael C. Carroll, Cynthia A. Lemere

**Affiliations:** Department of Neurology, Ann Romney Center for Neurologic Diseases, Brigham and Women’s Hospital, Boston, MA 02115, USA; Program in Cellular and Molecular Medicine, Boston Children’s Hospital, Harvard Medical School, Boston, MA, 02115, USA; Department of Neurology, Ann Romney Center for Neurologic Diseases, Brigham and Women’s Hospital, Boston, MA 02115, USA; Department of Neurology, Ann Romney Center for Neurologic Diseases, Brigham and Women’s Hospital, Boston, MA 02115, USA; Department of Neurology, Ann Romney Center for Neurologic Diseases, Brigham and Women’s Hospital, Boston, MA 02115, USA; Department of Neurology, Ann Romney Center for Neurologic Diseases, Brigham and Women’s Hospital, Boston, MA 02115, USA; Department of Neurology, Ann Romney Center for Neurologic Diseases, Brigham and Women’s Hospital, Boston, MA 02115, USA; Program in Cellular and Molecular Medicine, Boston Children’s Hospital, Harvard Medical School, Boston, MA, 02115, USA; Department of Neurology, Ann Romney Center for Neurologic Diseases, Brigham and Women’s Hospital, Boston, MA 02115, USA

**Keywords:** Complement C3 knockdown, aging, cognitive decline, synapse loss, hippocampal dysfunction

## Abstract

**Background:**

Complement component 3 (C3) is increasingly recognized for its role in neurodegenerative processes; however, its specific impact on age-related hippocampal dysfunction remains poorly understood. This study investigates the effects of inducible C3 knockdown in adulthood on hippocampal function using a novel mouse model.

**Methods:**

We developed a chimeric floxed C3 mouse line (*C3^fl/fl^*) and crossed it with *Rosa-26-Cre-ERT2+/−* mice, resulting in *C3^fl/fl^*; *Rosa-26-Cre-ERT2^+/−^* (*C3*iKO) mice that allow for global *C3* knockdown via Tamoxifen (TAM) administration at any age. Young adult female and male C3iKO mice were treated with TAM or corn oil (CO) as a control, to induce global C3 lowering in 4 cohorts of mice. Serum C3 levels were monitored throughout the lifespan for all cohorts. Other outcome measures varied by cohort and included behavior, C3 mRNA and protein levels in brain, C1q levels, immune gene expression in brain, gliosis, synaptic changes in hippocampus.

**Results:**

TAM treatment led to a sustained reduction in C3 levels in serum, liver, and brain tissues of *C3*iKO mice. Global C3 lowering was associated with reduced expression of *C1q, C4b, IFNa, IFNb,and APOE*, and increase expression of homeostatic genes *Tgfb1* and *Tgfbr1* in mouse brain one-year following TAM treatment. Notably, C3 lowering in adulthood conferred significant neuroprotection against age-related cognitive decline, which corresponded to increased hippocampal synaptic density and dendritic spine formation and increased pre-synaptic proteins in hippocampal synaptosomes. Moreover, long-term potentiation (LTP) impairments induced by Aβ-oligomers were rescued following *C3* knockdown, highlighting potential therapeutic implications.

**Conclusion:**

Our C3iKO mouse model was consistently effective in lowering C3 levels in the brain and periphery in mice. The findings reported here demonstrate that global C3 lowering in adulthood, after brain development, protected the brain against age-associated hippocampal dysfunction and cognitive decline, suggesting that complement modulation may provide a neuroprotective strategy against brain aging. The *C3iKO* model provides a valuable platform for understanding the role of complement C3 in age-related neurodegenerative conditions, including Alzheimer’s disease. Further studies are needed to better understand these neuroprotective effects in models of neurodegeneration and to assess the therapeutic potential of complement modulation in the brain.

## Background

Aging is a process by which cellular and tissue functions stop working properly, leading to progressive organ dysfunction[1]. Aging has been described as a prominent risk factor for the development of cardiovascular and neurodegenerative diseases[1, 2]. As a result of remarkable new technological advances in medicine over the past few decades, the number of elderly people continues to increase worldwide, leading to an increase in the prevalence of diseases such as Alzheimer’s Disease (AD), the most common form of dementia[3].

The complement system is a part of our innate immune system and is responsible, in part, for opsonizing and eliminating pathogens and damaged cells[4]. In the central nervous system (CNS), the complement cascade contributes to brain development. Complement molecules are expressed and secreted by microglia and astrocytes. They participate in the elimination of weak synapses and contribute to precise brain wiring, which is imperative in healthy brain development[5–8]. However, with aging, aberrant complement signaling may trigger detrimental synaptic pruning and contribute to neurodegenerative processes. During the aging process, complement is upregulated in the brain, plasma and other organs contributing to its pathogenesis at the sites of accumulation[9–13]. Some complement molecules have been reported to be essential to the normal brain development process. Complement components C1q and C3 remove pathogens and are critical for synapse elimination during brain development, aging, AD, schizophrenia, and other neurological diseases[14–28].

There are three well-defined complement pathways: classical, alternative and lectin[29, 30]. C3 is the common denominator of these pathways and plays a pathological role in CNS-associated diseases such as AD. Our group previously found that lifelong, germline C3-deficiency protected against synapse loss in the hippocampus during aging in wild-type mice[23]. While hippocampal CA3 synapse and neuron loss were observed in 16-month-old C57BL/6J WT mice, genetic knockout of *C3* protected against age-dependent decline in both synaptic puncta density and cognition[23]. Interestingly, the mechanism by which synapse loss is induced by Aβ oligomers, proximal synaptotoxins in AD[31], involves complement components C1q and C3 via microglia-mediated phagocytosis in which these molecules tag synapses and bind receptors on microglia, eliminating them in young mice injected with Aβ oligomers in the absence of Aβ plaques[26].Complement activation is crucial in the interaction between astrocytes and microglia, serving a protective function during the early stages of Alzheimer’s disease. However, in the later stages, it may contribute to synaptic deficits and the resulting cognitive decline[32]. We also reported that lifelong C3-deficiency was neuroprotective in aged, plaque-rich APP/PS1dE9 mice[27], which performed significantly better on cognitive tests than age- and gender-matched C3-sufficient APP/PS1dE9 mice and had partial sparing of hippocampal synapse and neuron loss despite having a significantly higher plaque load[4]. Furthermore, neuroinflammation (e.g., glial clustering and activation within plaques) was reduced in C3-deficient APP/PS1dE9 mice, suggesting that C3 may play a critical role in driving neuroinflammation, which facilitates synaptic decline.

The germline *C3*-knockout (*C3*KO) mouse model is useful for demonstrating the protective effects of C3 knockout in AD-like mouse models[14, 23–27], but it cannot be used to study whether the same neuroprotective effects can be achieved by inducing C3-deficiency after normal brain development. One group has generated a *floxed C3 IRES-tdTomato* reporter mouse which was crossed with a *Cre-Deleter* mouse on a C57BL/6 background to investigate the role of C3 in autoimmune diseases[33].

Here, we generated a chimeric *floxed C3* mice (*C3^fl/fl^*) and crossed them to *Rosa-26-Cre-ERT2^+/−^* mice to generate the *C3^fl/fl^*; *Rosa-26-Cre-ERT2^+/−^* (*C3iKO*) mouse line. *C3*iKO mice allow the induction of global C3 knockdown at any age via tamoxifen (TAM) injections. Our goal was to investigate whether C3 lowering in adulthood would protect hippocampal function in these mice later in life. TAM treatment in young mice led to consistent serum C3 lowering as they aged. We also found a C3 reduction in both protein and mRNA levels in the brain and liver of these mice. Further, global C3 lowering reduced C1q protein levels in the serum, brain and hippocampal synaptosomes over time in aged mice. Notably, aged *C3*iKO mice were protected against age-dependent synapse and dendritic spine loss and displayed a sparing of cognitive decline and LTP impairment induced by S26C Ab dimers. Together, these data suggest that targeting C3 signaling may be an effective therapeutic strategy for the aging brain, potentially slowing the rate of neurodegeneration. In addition, the beneficial effects of lowering C3 after the critical period of normal brain development suggest that targeting C3 in AD is likely to have some benefit as described in our accompanying paper.

## Methods

### Animals

Chimeric *C3* floxed mice (*C3^flfl^*) mice were generated on a C57BL/6J background using gene targeting by homologous recombination in embryonic stem (ES) cells. The *C3* targeting vector was purchased from EUCOMM (www.knockoutmouse.org/about/eucomm). The vector was transfected into the JM8A3 ES cell line, prepared from C57BL/6 mice, and has been used to construct several thousand germ-line strains by the EUCOMM consortium. *C3^fl/fl^* mice were crossed to *Rosa26-Cre-ERT2^+/−^*global inducible mice (Jackson Lab Stock: 008463) to generate *C3^fl/fl^;Rosa26-Cre-ERT2^+/−^*mice (*C3*iKO) which were bred and aged to generate the 4 cohorts used in these studies. Germline *C1qa*KO mice (Jackson Lab Stock: 031675) and *C3*KO (Jackson Lab Stock: 029661) were purchased as control mice for ELISAs. C57BL/6J mice wildtype (WT) mice (Jackson Lab Stock: 000664) were used as control mice for the LTP electrophysiology study. Mice were genotyped by Transnetyx Inc., using small pieces of ear tissue.

### Tamoxifen treatment

Tamoxifen (TAM) was dissolved in corn oil (CO) at 20 mg/ml concentration by shaking overnight at 37°C. After preparation, TAM solution was immediately used for the experiments. TAM was given via intraperitoneal (i.p.) injection at 75 mg TAM/kg body weight once every 24 hours for a total of 5 consecutive days. CO was i.p. injected into the vehicle control mice using the same schedule. A total of 4 cohorts of mice, including females and males, were used for the studies presented here as described in Table 1. Cohort A mice were treated with TAM or CO at 4-5 months (mo) of age and euthanized one year later at 16-17 mo of age; analyses included behavior, C3 and C1q levels in serum and brain, mRNA expression of complement, cytokine and other immune genes in brain, gliosis and synaptic puncta. Cohort B mice were treated with TAM or CO at 7-8 mo of age and euthanized 10 mo later at 17-18 mo of age; analyses included behavior, C3 and C1q levels in serum, gliosis, and C3, C1q and synaptic proteins in hippocampal synaptosomes. Cohort C mice were treated with TAM or CO at 2-3 mo of age, shortly after the completion of brain development, and euthanized at 2 mo later at 4-5 mo of age (Day 60 timepoint) or 5 mo later at 7-8 mo of age (Day 150 timepoint); analyses included serum C3 levels and brain mRNA expression of complement-related components, cytokines and homeostatic factors. Cohort D mice were treated with TAM or CO at 2-3 mo of age and euthanized 5 mo later at 7-8 mo of age for LTP electrophysiological studies or 13 mo later for serum C3 levels and dendritic spine analysis.

### C3 and C1q ELISA

To detect C3 levels (serum, brain and synaptosomes), plates were coated with goat-anti-mouse C3 antibody (MP biomedicals #55463). For its detection, the same C3 antibody was biotinylated according to the kit instructions (Thermo Scientific #90407). For serum analysis, the samples were diluted 50000x. As protein standard, purified mouse C3 (Complement Technology #M113) was used, starting at 1000 μg/ml following by serial dilutions. C1q levels were measured by coating the plate with rat anti-mouse C1q (Abcam 7H8 #11861) and detecting by rabbit-anti C1q antibody (Abcam JL-1 #ab71940) biotinylated in house as C3 antibody. To measure C1q and C3 protein levels in the brain and synaptosomes, mice were transcardially perfused with 1x PBS and brains were collected as fresh-frozen. Total protein from tissue was extracted using N-PER neuronal extraction buffer (Thermo Scientific #87792) containing protease and phosphatase inhibitors cocktail (Thermo Scientific #78430 and #78420) according to the manufactures’ s protocol. Total protein concentration was determined using a BCA kit (Thermo Scientific # J63283.QA). For both brain and synaptosomes, 100 μg of total protein was used in both C1q and C3 ELISAs. Afterwards, C3 and C1q ELISAs were followed according to the protocol described. Depending on the experiment, we used samples from *C3*KO and *C1*qKO mice as controls.

### Synaptosomes preparation

For synaptosomes preparation, all steps were performed at 4°C on ice, and all buffers contained protease and phosphatase inhibitor, as described for the kit’s instruction (Syn-PER, Thermo Scientific # 87793). Briefly, mice were euthanized by CO2 inhalation followed by decapitation. Afterwards, its hippocampi were rapidly dissected and homogenized in Syn-Per with approximately 1 ml of buffer per 100 mg of tissue. Samples were centrifuge at 1200 x g for 10 min. The resulting supernatant was then centrifuged at 15,000 x g for 20 min. Pellets (synaptosomes) were resuspended in the buffer as described in the kit protocol. Samples were stored long-term at −80°C, and protein concentration was quantified by BCA kit.

### Western Blot

Equal amounts of proteins (20 μg as determined using a BCA proteins assay) were resolved on 4-15% polyacrylamide precast gels (BioRad). The gel was electroblotted onto Odyssey Nitrocellulose membrane using 25 mM Tris, 192 mM glycine, 20% (v/v) methanol, pH 8.3, at 350 mA for 90 min at 4°C. Afterwards, membranes were blocked with commercial blocking solution (Intercept blocking buffer, Licor) for 1 h at room temperature. Primary antibodies included: anti-synaptophysin (Millipore, 1:200), anti-synapsin (Millipore, 1:200), anti PSD-95 (Millipore, 1:200) and GluN1 (Millipore, 1:200) and GAPDH (Abcam, 1:1000). The antibodies were diluted in the antibody dilution buffer (Licor) and incubated on the membranes overnight at 4°C. Afterwards, the membranes were incubated with secondary antibodies, anti-mouse or anti-rabbit, for Odyssey fluorescent detection. Membranes were incubated with IRDye-conjugated antibodies (1:10,000) (Licor) for 2 hours, washed and scanned in an Odyssey detector. Optical density was quantified on ImageJ. Original Western blot images are provided in Supplemental Figure 6.

### RNA extraction and quantitative RT-PCR

Total RNA was extracted from hemibrain using the PARIS kit system (Thermo Scientific, #AM1921) according to manufacturer’s instructions. Purity and amount of RNA were determined by the 260/280 nm absorbance ratio. Only preparations with ratios between 2.2 and 1.8 and no signs of RNA degradation were used. 1 μg RNA was used for cDNA synthesis using the High-Capacity cDNA Reverse Transcription Kit (Thermo Scientific #4368814). Expression of genes of interest was analyzed by PCR on an Applied Biosystems 7500 RT-PCR system using the Power SYBR kit (Thermo Scientific #4368577). Actin was used as an endogenous control. Cycle threshold (Ct) values were used to calculate fold-changes in gene expression using the 2^-DDCt^ method. In all cases, reactions were performed in 15 μl. A spreadsheet was created to show the primer sequences for genes in this study (Table 2).

### Immunohistochemistry and Immunofluorescent Immunostaining

Saline-perfused, hemibrains were fixed in 4% paraformaldehyde for 24 hours, and immunohistochemistry/immunofluorescence was performed as described previously. Fixed frozen sections (25 μm) were incubated with anti-GFAP (Dako 1:1000) and Iba-1(Wako 1:1000) overnight at 4°C. Afterwards, the sections were washed with 1x PBS and followed by incubation with biotinylated secondaries and Vector ELITE ABC kits (Vector laboratories #PK-6100). Furthermore, Vector DAB kit (Vector laboratories # SK-4100) was used to develop the reaction. For immunofluorescence, Alexa-Fluor 488 and Alexa-Fluor 568-conjugated secondary antibodies (1:2000) were incubated for 2 h at room temperature. Nuclei were stained with 4′,6-Diamidino-2-phenylindole dihydrochloride (DAPI) (Sigma #D9542) for 5 min. Slides were mounted with Entellan (Sigma #1.07960) or Prolong Gold Antifade (Thermo Scientific # P36930).

### Synaptic marker analysis

Free-floating sections (25 μm) were incubated overnight at 4°C with the following antibodies: anti-synaptophysin (Millipore, 1:200) and anti-homer-1 (Synaptic systems, 1:200). Analysis was performed as previously described. Confocal imaging was performed using Nikon Eclipse Ti camera with a Hamamatsu digital camera C10600 ORCA and 60x oil objective. Images were acquired with the following settings: 1 airy unit (AU) pinhole while holding constant gain and offset parameters for all sections and mice per experiment.

### Golgi Staining

Mice were euthanized by CO_2_ inhalation and followed by a perfusion with 1X PBS to remove the blood. Afterwards, the whole brain was carefully harvested and prepared for Golgi staining using the FD Rapid Golgi kit (FD Neurotechnologies) according to the manufacturer’s instructions. After staining, brains were sliced on a Leica Vibratome in 100 um-thick sections and developed for 10 minutes in solution according to the instructions. Images were acquired from the stained sections using a Nikon Eclipse E400 microscope. Each neuron was imaged under high magnification (100x). At least five neurons were selected each from hippocampal CA3 and dentate gyrus (DG) per mouse. The density of dendritic spines was measured with ImageJ software.

### Electrophysiology recordings

As described in, Cohort D mice were euthanized with Isoflurane at 7-8 months of age. Brains were quickly removed and submerged in ice-cold oxygenated sucrose-replaced artificial cerebrospinal fluid (ACSF) cutting solution (in mM) (206 sucrose, 2 KCl, 2 MgSO4, 1.25 NaH2PO4, 1 CaCl_2_, 1 MgCl_2_, 26 NaHCO_3_, 10 D-glucose, pH 7.4, 315 mOsm). Transverse slices (350 μm thickness) from the middle portion of each hippocampus were cut with a vibroslicer. After dissection, slices were incubated in ACSF that contained the following (in mM): 124 NaCl, 2 KCl, 2 MgSO_4_, 1.25 NaH2PO_4_, 2.5 CaCl_2_, 26 NaHCO_3_, 10 D-glucose, pH 7.4, 310 mOsm, in which they were allowed to recover for at least 90 min before recording. A single slice was then transferred to the recording chamber and submerged beneath continuously perfusing ACSF that had been saturated with 95% O_2_ and 5% CO_2_. Slices were incubated in the recording chamber for 20 min before stimulation under room temperature (~26°C).

For the record procedure, standard procedures were used to record field excitatory postsynaptic potentials (fEPSP) in the CA1 region of the hippocampus. A bipolar stimulating electrode (FHC Inc., Bowdoin, ME) was placed in the Schaffer collaterals to deliver test and conditioning stimuli. A borosilicate glass recording electrode filled with ACSF was positioned in stratum radiatum of CA1, 200~300 μm from the stimulating electrode. fEPSP in the CA1 region were induced by test stimuli at 0.05 Hz with an intensity that elicited a fEPSP amplitude 40-50% of maximum. Test responses were recorded for 30-60 min prior to beginning the experiment to assure stability of the response. Once a stable test response was attained, experimental treatments (Ab S26 dimers) were added to the 10 mL ACSF perfusate, and a baseline was recorded for an additional 30 min. To induce long-term potentiation (LTP), two consecutive trains (1 s) of stimuli at 100 Hz separated by 20 s were applied to the slices, a protocol that induced LTP lasting approximately 1.5 hr in wild-type mice of this genetic background. The field potentials were amplified 100x using an Axon Instruments 200B amplifier and digitized with Digidata 1322A. Data were sampled at 10 kHz and filtered at 2 kHz. Traces were obtained by pClamp 9.2 and analyzed using the Clampfit 9.2 program. LTP values reported throughout were measured at 60 min after the conditioning stimulus unless stated otherwise. Paired pulse facilitation of fEPSP was tested by two stimuli 50 msec apart.

### Preparation of synthetic Aβ (S26C dimers)

The S26C dimers vial (JPT #SP-Ab-24_0.5) was reconstituted according to the manufacturer’s instructions to a 50 μM concentration. For the experiments, we performed a time-course of the low-dose (from 5 to 50 nM) which led to LTP impairment in hippocampal slices from WT mice. Afterwards, 5 nM of S26C dimers were exposed to hippocampal slices from TAM- and CO-treated *C3*iKO mice, followed by electrophysiology recording.

### Behavioral tasks

#### Novel Object Recognition (NOR) and Displaced Object Recognition (DOR) tasks

NOR and DOR were carried out in an open field arena measuring 0.3 (*w*) ’ 0.3 (*d*) ’ 0.45 (*h*) m. Test objects were made of glass or plastic and had different shapes, colors, sizes, and textures. During behavioral sessions, objects were fixed with tape to the floor of the arena so the animals could not move them. None of the objects used in our experiments evoked innate preference. Training consisted of a 5 min session during which animals were placed at the center of the arena in the presence of two identical objects positioned in a straight line. The amount of time spent exploring each object was recorded.

Sniffing and touching the object were considered as exploratory behavior. The arena and objects were cleaned thoroughly between trials with 40% ethanol to eliminate olfactory cues. Two hours after training, animals were again placed in the arena for the test session.

In the NOR test session, one of the two objects used in the training session was replaced by a new one, whereas in the DOR test session, one of the objects was moved diagonally across the arena. Again, the amount of time exploring either the familiar or the novel/displaced object was measured by a trained researcher blinded to the identity of the experimental groups. Results were expressed through the discrimination index which consists of: (time with the novel object – time with the familiar object) / (time with the novel object + time with the familiar object). The analysis was performed using Ethovision software version 11 (Noldus Information Technology, Sterling VA).

#### Spatial Novelty Y-Maze (SNYM)

For this test, the maze was constructed out of acrylic and consist of three similar arms (45x15x30 cm): a “start” arm where animals are initially placed, a “familiar” arm and a “novel” arm, which it will remain blocked during the training session. The “novel” arm contains an object where it blocks the entrance into this place. Animals are placed facing away from the center of the maze in the “start” arm. The familiarization session consists of free exploration of the start arm and the familiar arm for 3 min. Afterwards, the mouse is placed into a holding cage for 2 min. During this time, the maze is cleaned and the object removed from the “novel arm”. The mouse is again placed in the maze, this time with access to all arms in the testing session. Allocation of the “familiar” and “novel” arms were counterbalanced among each experimental group. Trials lasted for 3 min, and center and nosepoints were recorded throughout each session. The experimental arena was cleaned with 20% ethanol solution after each trial. The analysis was performed using the Ethovision software version 11 (Noldus Information Technology, Sterling VA).

### Statistical analysis

Statistical analyses were performed using GraphPad Prism 8 software. Data are presented as mean ± the standard error of the mean (SEM). Two-tailed Student’s t tests (unpaired) were used when comparing 2 groups. Otherwise, a one-way or two-way ANOVA was used to determine statistical significance followed by a Tukey’s post-hoc test to specifically identify which groups were significantly different from each other. Operators were blinded to treatment groups for data collection and analyses in all experiments. The number of mice, p values and statistical tests used are indicated in the Figure Legends.

## Results

### Generation and characterization of *C3*^fl/fl^ and *C3^fl/fl^; Rosa26-cre-ERT2^+/^-* (*C3*iKO) mouse models

**Figure 1:**
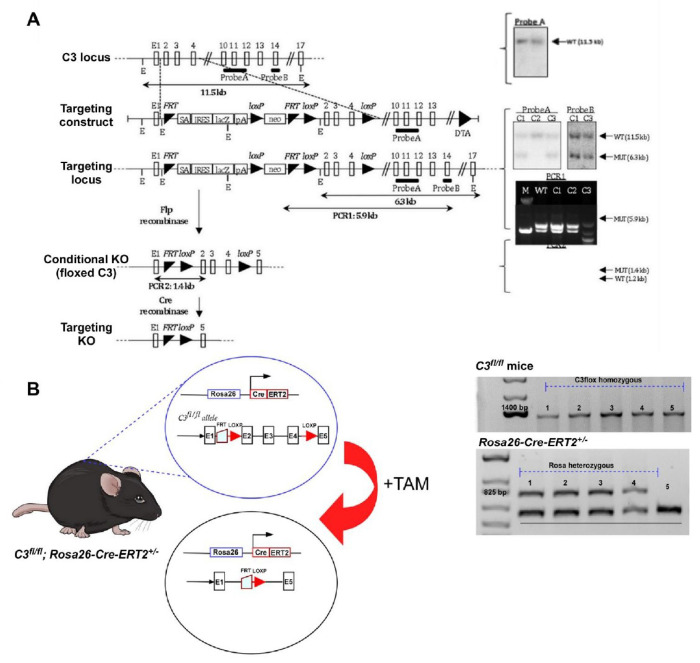
Characterization and generation of *C3^fl/fl^* and *C3^fl/fl^; Rosa26-Cre-ERT2^+/−^ (C3*iKO) mouse models. **(A)** Schematic diagram of partial *C3* allele; and Southern blot of WT JM8A3 following hybridization with Probe A on Eco RV digest showing expected WT allele, as well as the targeting construct and targeted allele. On the right, a southern blot of genomic DNA from neo-resistant ES clones following Eco RV digest and hybridization with Probes A (3 clones) or B (2 clones). Results confirm clones C1 and C3 bear the targeted allele. (Lower right panel) PCR analysis of ES cell clones reveal correct gene targeting. Floxed *C3* allele after *in vitro Flp* recombination to remove LacZ and the neo-cassette. PCR analysis of neo-sensitive clones confirmed removal of Lac-Z and the neo-cassette. Expected targeted allele after *in vivo* Cre recombination of *C3* gene. E:EcoRv; E1:exon1; SA;splice acceptor; pA:polyadenylation; WT: wildtype; MUT: mutant. **(B)** *C3^fl/fl^* and *Rosa26-Cre-ERT2+/−* mice were crossed with each other for 2 generations to produce *C3*^*fl/fl;*^ *Rosa26-Cre-ERT2+/−* (*C3iKO*) mice. On the right panel, PCR genotyping of the offspring revealed positive alleles for *C3*^*fl/fl*^ (~1400 bp; left panel) and *Rosa26-Cre-ERT2+/−* (~825 bp; middle panel). Mice 1-4 indicate the successful generation of the C3iKO mouse model.

To evaluate the effects of global C3 depletion in aged mice, we initially generated the *C3^fl/fl^*mouse model (Figure 1A). Using gene targeting by homologous recombination in embryonic stem (ES) cells, a null allele of *C3* was generated. Once we had the *C3* targeting vector (Figure 1A), it allowed us to generate mice with either a Lac-Z tagged allele or a *C3* floxed allele (Figure 1A). Next, we crossed our *C3 ^fl/fl^* mouse line with the *Rosa26-Cre-ERT2+-* mouse line for two generations to produce the experimental *C3^fl/fl^;Rosa26-Cre-ERT2+/−* (*C3*iKO) mice (Figure 1B) to allow global C3 lowering at any age.

For the studies presented here, we generated 4 cohorts of mice as shown in Table 1. Adult mice received once daily intraperitoneal (i.p.) injections of either tamoxifen (TAM) or corn-oil (CO) for 5 consecutive days as described in the Methods.

#### C3 levels were significantly reduced following tamoxifen administration

**Figure 2:**
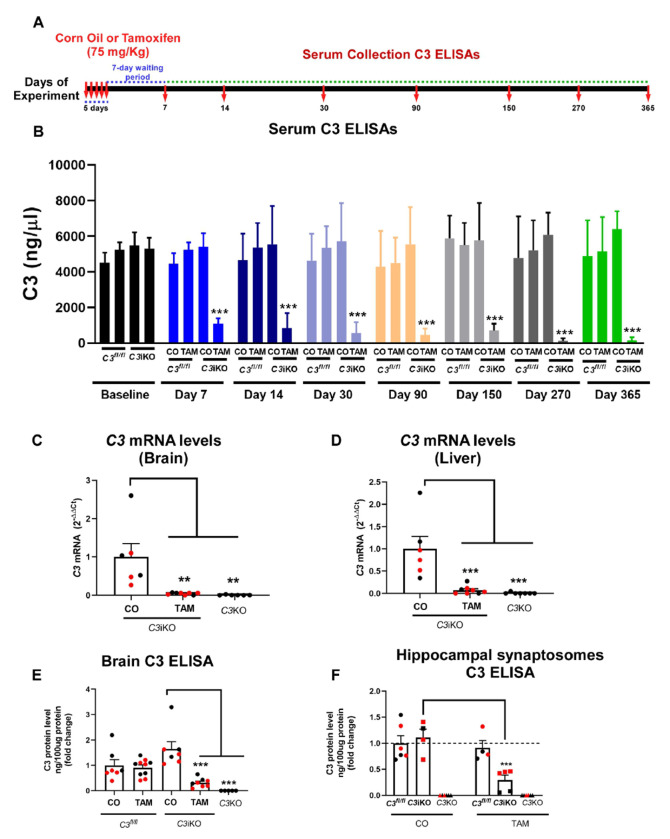
Tamoxifen treatment induced sustained C3 lowering in the serum, brain, liver in *C3*iKO mice. **(A)** Male and female mice in Cohort A were intraperitoneally (i.p) injected for 5 consecutive days with either corn oil (CO) or tamoxifen (TAM) at 4-5 mo of age and serum C3 protein levels were measured by ELISA at 7-, 14-, 30-, 90-, 150-, 270- and 365-days post-TAM treatment. Baseline C3 levels were obtained immediately prior to CO and TAM treatment. **(B)** TAM treatment in adult *C3*iKO mice showed consistent lowering of serum C3 levels across all time points. Data are expressed as means ± the standard error of the mean (SEM). Two-way ANOVA followed by a Tukey post-hoc test. *** p<0.001. N= 6-10 mice per group. TAM=tamoxifen; CO= corn oil. **(C-D)** *C3* mRNA levels in the brain **(C)** and liver **(D)** in 16-17 mo-old *C3*iKO mice from Cohort A 1 year after the last injection of tamoxifen. Values shown are mean + SEM fold-change expression in *C3*iKO + TAM mice compared to *C3*iKO + CO and germline *C3*KO mice. One-way ANOVA followed by a Tukey post-hoc test. ** p<0.01, *** p<0.001. N= 4-5 mice per group. **(E)** C3 protein levels in the brains of Cohort A *C3*iKO mice measured by ELISA at 16-17 mo of age. Values shown are mean ± SEM. Two-way ANOVA followed by a Tukey post-hoc test. *** p<0.001. N= 6-10 mice per group. **(F)** C3 protein levels in hippocampal synaptosomes of *C3*iKO, *C3*^fl/fl^ and *C3*KO mice measured by ELISA at 17-18 mo of age (Cohort B) after TAM or CO treatment at 7-8 mo of age. Values shown are mean ± SEM. Two-way ANOVA followed by a Tukey post-hoc test. *** p<0.01. N= 4-6 animals per group. In all panels, dots correspond to individual mice (red for female, and black for male. TAM= tamoxifen; CO= corn oil.

Systemic complement C3 is synthesized mainly by the liver and can be detected in the serum. Therefore, serum was collected at various timepoints after treatment to perform C3 protein ELISAs. This enabled us to evaluate whether efficient recombination of the Cre recombinase occurred, thereby reducing serum C3 levels. We detected consistent and sustained C3 lowering in the serum up to one year in aged *C3*iKO mice that received TAM at 4-5 months (mo) of age compared to those that received CO (Cohort A; Figure 2A-B). As expected, TAM injections had no effect on serum C3 levels in *C3^fl/fl^* mice. Aged *C3*iKO mice treated with TAM at 7-8 mo of age showed a similar and consistent reduction in serum C3 levels from 7 to 320 days post-TAM treatment (Cohort B; Supplemental Figure 1). Cohort C mice treated with TAM at 2-3 mo of age and euthanized 2 or 5 mo later showed significant C3 lowering at both timepoints (Supplemental Figure 3A). Cohort D mice treated with TAM or CO at 2-3 mo of age also showed sustained C3 lowering over 395 days (Supplemental Figure 5). By the end of each study, we detected a significant reduction of 88-98% reduction in TAM-treated *C3*iKO mice but no reduction in control mice.

Next, we assessed *C3* mRNA and protein levels in the brains of 16–17 mo-old *C3*iKO mice from Cohort A, one year after the last injection of TAM. *C3* mRNA levels in the brain and liver were measured by qPCR and were significantly reduced by >90% in the TAM-treated mice compared to the CO-treated mice (Figure 2C-D). Remarkably, these reductions were similar to the germline *C3*KO mice that were used as controls in our experiments. To assess the C3 protein levels, hemibrain tissues were homogenized and analyzed by ELISA. C3 protein levels were significantly reduced by ~80% in the brains of the TAM-treated *C3*iKO mice but not in the control mice (CO-treated *C3*iKO and *C3^fl/fl^* mice treated with either TAM or CO) (Figure 2E). Furthermore, we isolated synaptosomes, structures that contain functional synaptic proteins from neuronal tissue, from the hippocampi of 17–18 mo-old *C3*iKO mice in Cohort B that were treated with TAM or CO 10 months earlier. C3 protein levels in synaptosomes were significantly reduced by ~70% in TAM-treated *C3*iKO mice when compared to the CO-treated *C3*iKO mice (Figure 2F). *C3* mRNA and protein levels were undetectable in the germline *C3*KO mice, as expected. Therefore, TAM treatment effectively reduced the overall *C3* expression in the liver and brain, and C3 protein levels in the serum, brain and hippocampal synaptosomes in aged *C3*iKO mice.

#### Global C3 lowering in adult mice alleviated age-associated cognitive impairment

**Figure 3:**
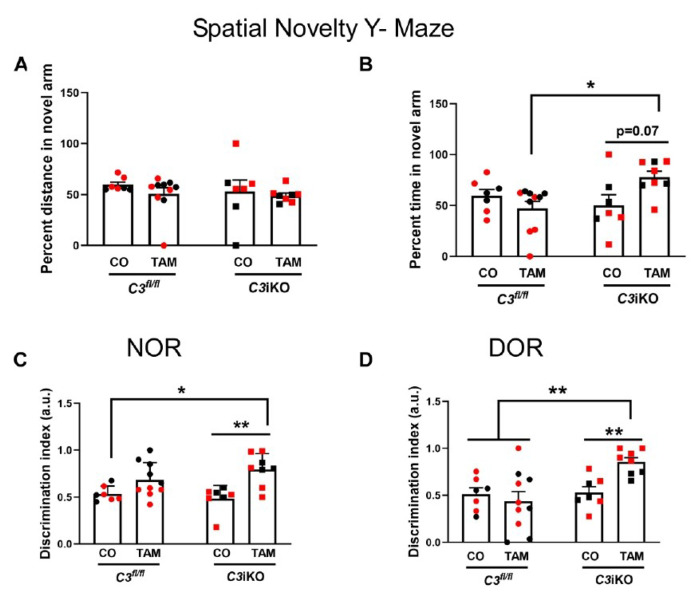
Global C3 lowering spared cognitive impairment aged mice. *C3*^*fl/fl*^ and *C3*iKO mice were treated with TAM or CO at 4-5-months-old (Cohort A). Behavioral tests were performed when the mice reached 16-17 mo of age and included **(A-B)** Spatial Novelty Y-Maze (SNYM) and **(C-D)** Novel and Displaced Object Recognition tasks (NOR and DOR, respectively). **(A)** shows the percent distance travelled in the novel arm of the SNYM, while (B) shows the percent time spent in the novel arm of the SNYM. **(C-D)** show the discrimination index for the NOR and DOR, respectively. In all panels, dots correspond to individual mice (red for female, and black for male). Two-way ANOVA followed by a Tukey post-hoc test. * p<0.05, ** p<0.01. N= 7-8 mice per group. TAM= tamoxifen; CO= corn oil.

Age-related and hippocampal-dependent memory decline have been extensively reported in rodents,. Our group has previously shown that loss of hippocampal synapses and neurons was associated with age-related learning and memory deficits. Overall, germline *C3*-deficiency resulted in a sparing of age-related cognitive decline when compared to *C3*-sufficient WT mice. Here, we performed a battery of behavioral experiments to evaluate whether C3 lowering in adult mice, after brain development, would protect against age-related cognitive decline. *C3*iKO mice at 16-17 mo of age (Cohort A) were subjected to the Spatial Novelty Y-Maze (SNYM), a task designed to evaluate spatial memory in mice. While there was no statistical difference in the distance traveled in the novel arm (Figure 3A), the *C3*iKO TAM-treated mice showed a strong trend (p < 0.07) of spending more time in the novel arm suggesting a modest improvement in spatial memory when compared to the CO-treated mice (Figure 3B). Remarkably, 17-18 mo-old *C3*iKO mice (Cohort B), who were treated with TAM later in life, performed significantly better on this test in both the percent distance and percent time spent in the novel arm compared to the CO-treated control mice and, performed similarly to germline *C3*KO mice (Supplemental Figure 2A-B).

Next, we assessed the novel object recognition (NOR) task in 16-17 mo-old *C3*iKO mice (Cohort A) and found that C3 lowering in the TAM-treated *C3*iKO mice resulted in a significant improvement in cognitive function (Figure 3C). We further explored the protective role of global C3 lowering within the same group of mice in a hippocampal-dependent versionof the displaced object recognition (DOR) task. TAM-treated *C3*iKO mice had a significantly higher discrimination index than CO-treated control mice indicating that C3 lowering in adulthood protected against age-dependent memory decline (Figure 3D). In addition, 17-18 mo-old *C3*iKO mice (Cohort B) who were treated with TAM later in life also had significantly higher discrimination index levels, confirming better cognitive performance in both the NOR and DOR tasks (Supplemental Figure 2C-D). These findings from 2 studies demonstrate that the C3 lowering in adult *C3*iKO mice protected against age-associated memory deficits and cognitive impairment.

#### Global C3 lowering altered immune gene expression in mouse brains

**Figure 4:**
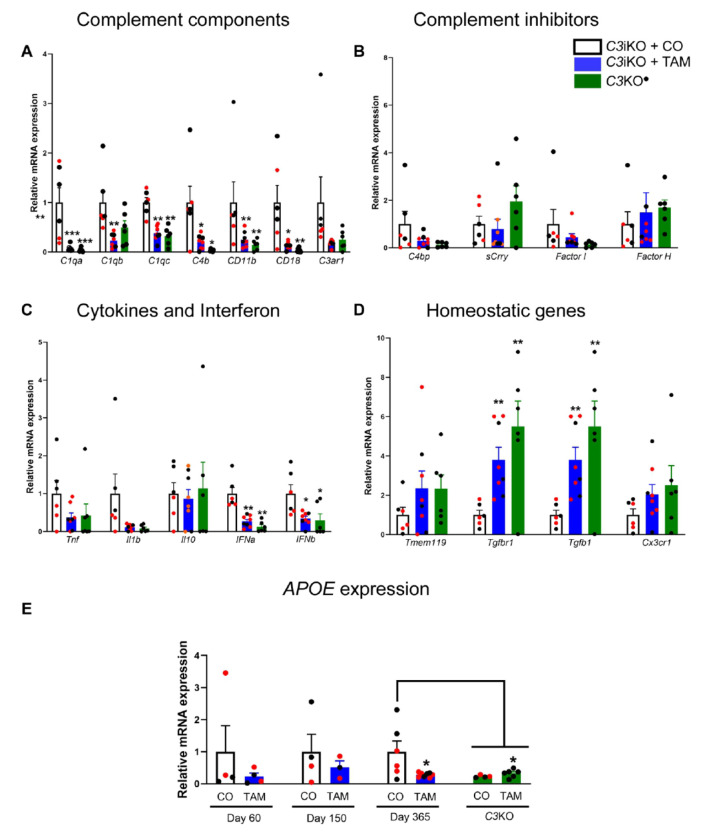
C3 lowering in adulthood altered immune gene expression in the brains of aged *C3*iKO mice. Gene expression measured by qRT-PCR in the brains of 16-17 -old C3iKO mice that were treated with TAM at 4-5 months of age (Cohort A). **(A)** qRT-PCR results for complement components C1q (*C1qa, C1qb* and *C1qc*), *C4b*, CR3 (*CD11b* and *CD18*) and C3aR1 (*C3ar1*). **(B)** qRT-PCR results for complement inhibitors *C4bp, sCrry, Factor I* and *Factor H*. **(C)** qRT-PCR results for cytokines *Tnf, IL1b, IL10* and interferons *IFNa* and *IFNb*. **(D)** qRT-PCR results for homeostatic genes *tmem119, tgfbr1, tgfb1* and *cx3cr1*. **(E)** Brain *APOE* mRNA levels on Days 60, 150 and 365 following TAM or CO treatment in Cohort A mice. *APOE* mRNA levels were significantly reduced in the TAM-treated *C3*iKO mice 1-year post-TAM treatment and similar to those of germline *C3*KO mice. Values shown are mean ± SEM fold-change expression in *C3*iKO + TAM compared to *C3*iKO + CO mice. One-way ANOVA followed by a Tukey post-hoc test. * p<0.05, ** p<0.01, *** p<0.001. N= 4-5 animals per group. In all panels, dots correspond to individual mice (red for female, and black for male). As indicated in the figure, white bars correspond to the CO-treated *C3*iKO mice and blue bars correspond to the TAM-treated *C3*iKO mice. The green bars represent *C3*KO mice.

To study the effects of C3 lowering and its potential to induce changes in gene expression in aged mice, we performed qRT-PCR in brains of 16-17 mo-old *C3*iKO mice (Cohort A) treated with TAM at 4-5 months of age for a series of immune genes known to be upregulated or downregulated during aging. As mentioned earlier, *C3* mRNA levels in the brain remained reduced for at least one-year post-TAM treatment the (Figure 2B). We found a significant reduction in the expression of complement components, e.g., *C1qa, C1qb, C1qc, C4b, CD11b and CD18* one-year post-TAM treatment, similar to levels in germline *C3*KO mice (Figure 4A). Surprisingly, we detected time-dependent changes in some of these genes in a small sub-study in which *C3*iKO mice were treated with TAM or CO at 2-3 mo of age and examined 2 months (Day 60) or 5 months (Day 150) post-treatment (Cohort C; Supplemental Figure 3). On Day 60, *C1qa* mRNA expression was significantly elevated in the TAM-treated *C3*iKO mice compared to CO-treated controls, even though the levels of mRNA *C3* remained low (Supplemental Figure 3B). On Day 150, *C1qa* mRNA expression dropped and was similar between TAM- and CO-treated C3iKO mice (Supplemental Figure 3B). These findings indicate the possibility of a time-dependent compensatory mechanism between astrocytes and microglia which could explain this intriguing data. Consistent with our findings, Guttikonda and colleagues previously demonstrated an increase in C1q protein levels upon C3 knockdown in tricultures with microglia, astrocytes and neurons. We did not observe any changes in genes related to the inhibition of the complement cascade in Cohort A mice one-year post-treatment (e.g., *C4bp*, *Scrry, Factor I and Factor H*) (Figure 4B). However, in the Cohort C sub-study, *mCr2* expression was significant reduced at Day 60 and *Factor I* expression was significantly reduced at Day 150 indicating time-dependent changes (Supplemental Figure 3C).

While no significant changes were seen for cytokine genes *Tnf, IL-1b* and *IL-10* in Cohort A TAM-treated mice, interferon *IFN1a* and *IFN1b* expression was significantly reduced one-year post-treatment compared to CO-treated mice and was similar to that of germline *C3*KO mice (Figure 4C). It has been reported that the interferon response drives neuroinflammation in AD-like mouse models through complement C3-dependent synapse elimination. Conversely, the study showed a rescue of synapse loss and a reduction of microglia activation after blocking IFN signaling. In our sub-study (Cohort C) mice, we detected a significant reduction in *IFNb* expressionon Day 150 but not on Day 60 (Supplemental Figure 3D). These results suggest that long-term complement C3 inhibition led to the reduction of *IFN* expression.

The loss of homeostatic function in microglia and how it contributes to the pathogenesis of AD has been reported. We asked whether global C3 lowering causes changes in homeostatic gene expression, which are impaired in microglia in AD, within the brains of aged mice. In the *C3*iKO TAM-treated mice in Cohort A, we found a significant increase in the expression of *Tgfbr1* and *Tgfb1* genes, similar to that seen in *C3*KO mice, compared to CO treated mice (Figure 4D). Interestingly, we detected a reduction in the expression of several homeostatic genes in the brain, including *Tmem119, Tgfbr1, Tgfb1, and P2ry12* at Day 150 in 7-8 mo-old C3iKO mice treated with TAM 5 months earlier (Cohort C; Supplemental Figure 3D), which emphasizes a dynamic modulation of homeostatic genes over time when inducing C3 lowering in *C3*iKO mice.

#### C3 lowering reduces *APOE* gene expression in aged mice

Apolipoprotein-E (ApoE) has been implicated in several diseases, including AD and cardiovascular disease such as atherosclerosis. In AD, the *APOE* variant e4 (*APOE*ε4) has been described as the largest risk allele for AD. Although the specific mechanisms of action in the pathogenesis of AD remain elusive, some studies have proposed a possible interaction between ApoE and some complement components, such as C1q and C3. In human AD brains, an interaction between *APOEε4* and cerebrospinal fluid (CSF) C3 was reported, suggesting an interrelationship between *APOE*ε4 and C3 that may contribute to neurodegenerative processes in AD. This prompted us to evaluate whether global C3 lowering would affect *APOE* mRNA expression in our mouse model. To address this question, we performed qRT-PCR for *APOE* in the brain samples from Cohort A *C3*iKO mice. C3 lowering significantly reduced the mRNA *APOE* levels 1-year post-TAM treatment (Figure 4E). Interestingly, aged germline *C3*KO mice exhibited the same reduction of *APOE* expression in the brain samples in both CO- and TAM-treated groups. These results suggest that the early induction of C3 lowering modulated *APOE* expression in aged mice.

#### Global C3 lowering reduced of C1q levels in aged mice

**Figure 5.**
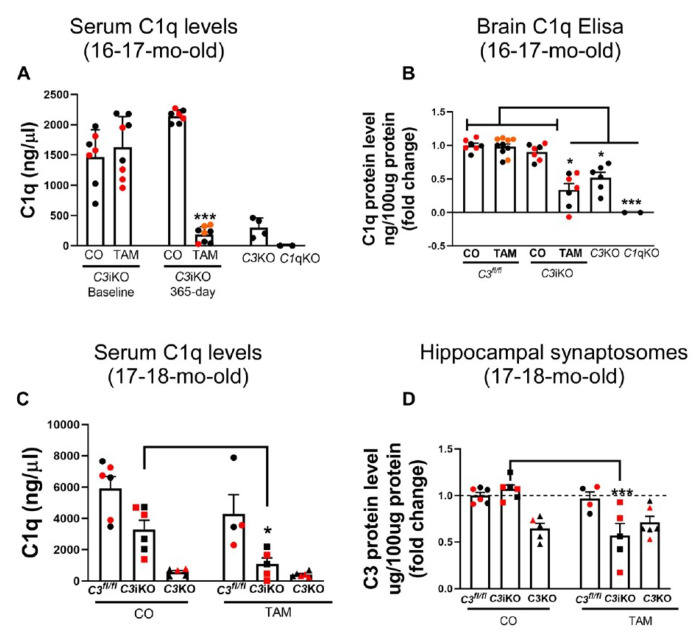
Global C3 lowering reduced C1q levels in serum, brain and hippocampal synaptosomes. **(A)** Serum C1q levels measured by ELISA in 16-17 mo-old mice (Cohort A) 1-year after the last TAM injection. Germline C3KO and *C1qKO* mice were included as controls. Data are expressed as means ± SEM. Two-way ANOVA followed by a Tukey post-hoc test. *** p<0.001. N= 2-8 animals per group. **(B)** Hemibrain C1q protein levels measured by ELISA in 16-17 mo-old mice (Cohort A). Data are expressed as means ± SEM. Two-way ANOVA followed by a Tukey post-hoc test. * p<0.05, *** p<0.001. N= 2-8 animals per group. **(C)** Serum C1q levels measured by ELISA in 17-18 mo-old mice (Cohort B), 10-months after the TAM last injection. Data are expressed as means ± SEM. Two-way ANOVA followed by a Tukey post-hoc test. * p<0.05. N= 4-6 animals per group. **(D)** C1q protein levels in hippocampal synaptosomes of Cohort B 17-18 mo-old mice. Data are expressed as means ± SEM. Two-way ANOVA followed by a Tukey post-hoc test. *** p<0.001. N= 4-6 animals per group. In all panels, dots correspond to individual mice (red for female, and black for male). TAM= tamoxifen; CO= corn oil.

Synapse loss has been described as an early event in the pathogenesis of AD, occurring up to 20 years prior to the onset of memory loss. Aging also leads to synaptic loss. Studies in rodent models have implicated the classical complement pathway in pathological synaptic loss processes. C1q is deposited on synapses to induce their elimination. Previous studies have shown that synapse loss is reduced or abolished when C1q is knocked out or blocked with a specific antibody in AD-like mouse models. We investigated whether global C3 lowering changes C1q levels in our *C3*iKO mice. As previously noted, C3 lowering caused a decrease in *C1q* mRNA 1-year post-TAM treatment in 16–17 mo-old mice (Cohort A; Figure 4A). C1q ELISA revealed a significant reduction in serum C1q protein levels in Cohort A mice one-year after TAM treatment, with similar levels in germline *C3*KO and *C1q*KO mice (Figure 5A). C1q ELISA of brain homogenates also showed a significant reduction in C1q protein levels in the brains of *C3*iKO TAM-treated mice, *C3*KO and *C1q*KO mice compared to CO-treated *C3*iKO and CO- and TAM-treated *C3^fl/fl^* mice (Figure 5B). We further analyzed C1q protein levels in the serum and hippocampal synaptosomes in Cohort B 17–18 mo-old mice and detected significantly reduced C1q protein levels in serum (Figure 5C) and hippocampal synaptosomes (Figure 5D) 10 months after TAM treatment. Remarkably, a C1q levels were consistently reduced in the germline *C3*KO mice, further confirming the data from our *C3*iKO mice. Our data corroborate a report which showed a reduction in C1q levels in *C3*KO mice in an age-dependent manner.

#### C3 lowering did not alter microglia or astrocyte reactivity or number

**Figure 6:**
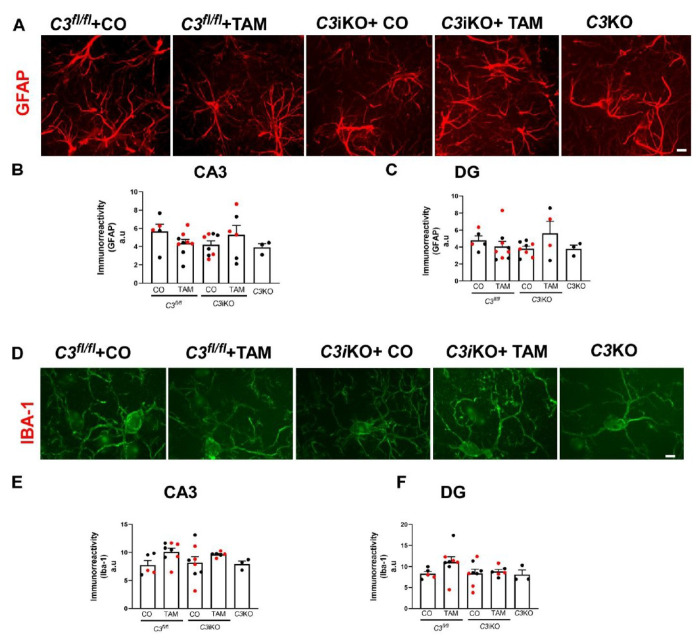
Global C3 lowering did not affect microglia and astrocytes in aged *C3*iKO mice. **(A)** Images represent GFAP (astrocyte marker) immunofluorescence in the hippocampi of 16-17 mo-old *C3*^*fl/fl*^, *C3*iKO and *C3*KO mice treated with either CO or TAM at 4-5 mo of age (Cohort A). Scale bar: 10 μm. **(B-C)** Quantification of GFAP fluorescence intensity in both hippocampal CA3 and dentate gyrus (DG) in Cohort A mice. **(D)** Images represent Iba-1 (microglia/macrophage marker) immunofluorescence in hippocampi of Cohort A mice. Scale bar: 10 μm. **(E-F)** Quantification of Iba-1 fluorescence intensity in both hippocampal CA3 and DG in Cohort A mice. Two-way ANOVA followed by a Tukey post- hoc test. N= 3-9 animals per group. In all panels, dots correspond to individual mice (red for female, and black for male). TAM= tamoxifen; CO= corn oil.

Microglia and astrocytes interact via contact-dependent and secreted factors to coordinate their proper function in the context of health and disease. In brain, astrocytes are the main source of complement component C3 synthesis. In addition, microglia can also produce C3 when they lose their homeostatic function under pathological conditions. Several studies have reported that microglia and astrocytes become more reactive with age. Therefore, we investigated whether complement *C3* lowering could modulate the amount of immunoreactivity or the number of astrocytes and microglia in aged *C3*iKO mice (Cohort A: 15-16 mo-old) (Figure 6). Surprisingly, we did not find any significant changes in either GFAP (astrocytic marker) (Figure 6A–C) or Iba-1 (microglia/macrophage marker) (Figure 6 D–F) reactivity in the hippocampi of *C3*iKO TAM-treated mice. Furthermore, we observed no significant changes in hippocampal glial immunoreactivity or the number of glial cells in Cohort B mice at 17-18 mo of age (Supplemental Figure 4). These findings suggest that global C3 lowering did not affect microglia and astrocyte labeling or number within a given region of the hippocampus.

#### C3 lowering protected against synapse and dendritic spine loss and rescued LTP impairment induced by Aβ-oligomers in aged mice

**Figure 7:**
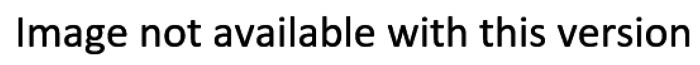
Global C3 lowering in adulthood protected synapses and hippocampal function during aging. **(A)** Representative confocal images for Synaptophysin (Syp; pre-synaptic marker) and Homer-1 (post-synaptic marker) in the hippocampi of Cohort A CO-treated and TAM-treated C3iKO mice. Synapses appear as yellow co-localized puncta. Scale bar: 10 μm. **(B-C)** Number of co-localized Syp/Homer-1 puncta in both hippocampal CA3 **(B)** and DG **(C)** of 16-17 mo-old mice following TAM or CO treatment at 4-5 mo of age. Two-way ANOVA followed by a Tukey post-hoc test. * p<0.05. N= 2-6 animals per group. **(D)** Images represent Golgi staining of dendritic spines in the brains of C57BL/6J WT, *Rosa-26-Cre-ERT2*^*+/−*^ and *C3*iKO mice that were treated with TAM or CO at 2-3 mo of age and euthanized at 15-16 mo of age (Cohort D). Magnification: 100X **(E-F)** Graphs represent the dendritic spine density in apical dendrites of neurons from the CA3 and DG areas of the hippocampi of Cohort D mice. Each symbol corresponds to the mean of three independent 10 μm segments per neuron, 5 neurons per mouse (N = 4–6 mice/group). Two-way ANOVA followed by a Tukey post-hoc test. * p<0.05, p<0.01. N= 4-6 animals per group. **(G)** Western blotting and quantification of pre-synaptic (Synaptophysin and Synapsin) and post-synaptic proteins (PSD-95 and GluN1) in hippocampal synaptosomes isolated from *C3*iKO and *C3*KO mice that were treated with TAM or CO at 7-8 mo of age and euthanized 10 mo later (Cohort B). One-way ANOVA followed by a Tukey post-hoc test. *p<0.05. N= 3-6 animals per group. In all panels, dots correspond to individual mice (red for female, and black for male). TAM= tamoxifen; CO= corn oil. **(H)** Field excitatory post-synaptic potential recordings in acute hippocampal slices from 7-8 mo-old WT mice exposed to vehicle or 5-50 nM S26C dimers (N = 8-9 slices from 4 mice/group). **(I)** Field excitatory post-synaptic potential recordings in acute hippocampal slices from 7-8 mo-old *C3*iKO treated with CO or TAM at 2-3 mo of age (Cohort D). Hippocampal slices were exposed to 5 nM S26C dimers in artificial cerebral spinal fluid (CSF) (N = 9–10 slices from 5 mice/group).

Synapse loss has been described as a hallmark of several neurological diseases, including AD. Aging-related changes in the brain include synapse loss, with several studies showing a significant decrease in the number of hippocampal synapses and dendritic spines in WT mice with aging. Previously, we showed that germline C3-deficiency in aged mice protected against synapse loss in the hippocampus. Here, we investigated whether C3 lowering in adult mice could offer similar protection against age-dependent synapse loss in the hippocampus in *C3*iKO mice. Synaptic puncta were quantified by measuring the co-localization of a presynaptic marker (Synaptophysin) and a postsynaptic marker (Homer-1) in Cohort A mice that were treated with TAM or Co at 4-5 mo of age and euthanized 12 mo later (Figure 7A). C3 lowering by TAM treatment, described earlier (Figure 2), protected against the age-associated loss of hippocampal CA3 synaptic puncta when compared to CO-treated control mice (Figure 7B). However, only a non-significant trend for protection against synapse loss was evident in the hippocampal dentate gyrus (DG) (Figure 7C). In both CA3 and DG, synaptic puncta were highest in germline *C3*KO mice, confirming our earlier findings. Synaptic puncta were unchanged by TAM treatment in the *C3^fl/fl^* control mice and were similar to the CO-treated *C3*iKO mice (Figure 7B–C), confirming that C3 lowering protected synaptic puncta.

To further evaluate the effect of global C3 lowering on age-dependent synapse loss, dendritic spine density analysis was performed using the Golgi-Cox staining method in hippocampal CA3 and DG regions in Cohort D mice that were treated with TAM or CO at 2-3 mo of age and euthanized at 15-16 mo of age. WT and *Rosa26Cre-ERT2+/−* mice were also treated with TAM or CO as controls. Serum C3 levels were significantly reduced by TAM treatment in *C3*iKO mice but not in WT mice nor *Rosa25Cre-ERT2+/−* control mice (Supplemental Figure 5). Neurons from the stratum radiatum in CA3 and stratum moleculare in DG were analyzed. Dendritic spines were significantly lower in both WT and *Rosa26-Cre-ERT2^+/−^* mice controls treated with CO or TAM, and in CO-treated *C3*iKO mice (Figure 7D–F). Global C3 lowering in TAM-treated *C3*iKO mice resulted in significant protection against dendritic spine loss in the CA3 region (Figure 7E), and a strong trend (p<0.06) for protection in the DG region of the hippocampus (Figure 7F). Thus, global C3 lowering in adulthood protected against age-associated loss of dendritic spines.

In addition to synaptic markers, we investigated the biochemical levels of several pre- and post-synaptic proteins in isolated hippocampal synaptosomes from Cohort B *C3*iKO mice treated with TAM or CO at 7-8 mo of age and euthanized 10 mo later. Interestingly, we detected a significant increase in the protein levels of presynaptic markers (Synaptophysin and Synapsin) but no changes in the levels of postsynaptic markers (PSD-95 and GluN1) in TAM-treated *C3*iKO mice compared to CO-treated *C3*iKO mice (Figure 7G).

Aβ-oligomers have been characterized as one of the main molecules that act as prime synaptotoxic agents in AD. A synthetic Aβ_40_ peptide, in which the serine 26 was mutated to cysteine (S26C), forms dimers that can perturb normal synapse physiology. We hypothesized that global C3 lowering would protect against the long-term potentiation (LTP) impairment induced by the S26C dimers. To test this hypothesis, we first determined the lowest concentration of the S26C dimers that would induce a LTP impairment. WT hippocampal slices were exposed to 5-50 nM doses of S26C. All doses, including a low dose (5 nM) of the S26C dimers, blocked LTP induction (Figure 7H). Next, we isolated the hippocampi of 7-8 mo-old *C3*iKO mice that were treated with TAM or CO at 2-3 mo of age to perform an electrophysiology experiment. S26C dimers were added which induced a LTP impairment in hippocampal slices of CO-treated mice (black dots) (Figure 7I). However, the same concentration of S26C dimers failed to induce a similar LTP impairment in the hippocampal slices of TAM-treated mice (red dots) (Figure 7I). These results suggest that global C3 lowering fully prevented the impairment of hippocampal LTP by soluble S26C Aβ dimers.

## Discussion

In our current work, we demonstrate that a global reduction of complement C3, a central molecule in the innate complement immune system responsible for mediating the removal of opsonized pathogens and debrisand for promoting synapse elimination through microglia-mediated phagocytosis, during adulthood prevented age-dependent memory decline and alleviated synapse loss in aged mice. Furthermore, conditional knockdown of C3 prevented LTP impairment induced by soluble Aβ oligomers in adult mice.

Previous studies, including from our group have reported cognitive improvements from germline complement *C3*-deficiency (*C3*KO) in aged and AD-like mouse models. During aging, several complement molecules, including C3, are elevated in both human and mouse brains. Furthermore, elevated levels of complement protein in the CSF of AD patients have been observed and were correlated with amyloid and tau deposition and cognitive decline relative to reduced brain volume. Notably, C3, which plays a central role during normal brain development, along with another important molecule, C1q, coordinate structural plasticity and functional homeostasis of synapses in the developing brain. Therefore, targeting these molecules through the generation of germline knockout mouse models has been useful to better understand their role in inadequate synaptic pruning, which in turn could hinder brain development and contribute to pathogenesis later in life. However, germline knockout mice have limited relevance to reasonable therapeutic strategies in humans as treatments would likely be started well after birth. Newer technologies allow for knocking down various genes conditionally, after normal brain development. This permits the study of how global reduction of specific complement molecules, such as C3, later in life impacts important neurodegenerative processes in AD.

To that end, we generated a *C3^fl/fl^* mouse model to target the complement C3 molecule. We crossed *C3^fl/fl^*mice with *Rosa-26-Cre-ERT2^+/−^*to generate inducible global C3 conditional knockout mice (*C3*iKO), in which C3 can be suppressed by TAM treatment at any age. This model allowed us to determine whether C3 suppression after brain development is completed but before the initiation of age-associated hippocampal dysfunction and cognitive decline, would be protective.

To test the efficacy of our model, we treated 4 cohorts of mice with TAM or CO in adulthood and examined them at various timepoints thereafter. We showed that TAM treatment resulted in a consistent global C3 lowering in the serum, liver, and brain at progressive time periods after the last injection. Liver, is the main sources of complement in the human bodyand TAM treatment efficiently lowered C3 mRNA and protein in the liver of the C3iKO mice.

Several reports have previously demonstrated age-related deficits in hippocampus-dependent learning and spatial memory tasks . Here, we found that global C3 lowering in adulthood protected aged mice against typical age-dependent memory decline. While germline C3-deficiency protected against cognitive impairment in the Water T-Maze and Contextual Fear Conditioning, *C3*iKO and *C3*KO mice performed better on the Spatial Novelty Y-Maze and Novel- and Displaced- Object Recognition tasks in this current study.

Microglia and astrocytes coordinate important immunologic responses within the CNS. These glial cells play a central role in the production of central molecules C1q and C3 in brain. In a diseased brain state, these classical complement components are produced in excess by reactive astrocytes and microglia, resulting in microglia-mediated synapse elimination. In our study, we show a transient response of *C1q* mRNA levels in the brain upon global C3 depletion. The expression of *C1q* increased initially but was significantly decreased at a later timepoint. This suggests a possible interference in the crosstalk between microglia and astrocytes, as we did not observe a significant change in glial cell morphology, although we observed significant changes in mRNA expression of cytokines such as *Tnf, Il1* and *IFN*. It has been reported that *C3* knockdown in a triculture model of microglia, astrocytes, and neurons led to increased C1q protein levels. The authors hypothesized that deleting C3 disrupted homeostasis, resulting in higher levels of C1q. This rationale might apply to our *in vivo* results as well. Another study demonstrated that germline *C3* deficiency leads to C1q reduction in the serum of mice in an age-dependent manner which is consistent with our observations.

The impact of aging on microglia has been controversial. Some previous studies have reported that aging itself is associated with only minor morphological changes in microglia. Another report suggests that an activated microglia phenotype earlier in life could alter cells and cause a change in microglial phenotype later in life. This might be a possible explanation for why we did not observe any microglial and astrocytic activation in the aged mice. Homeostatic microglia express genes responsible for homeostasis, and these genes are downregulated in a diseased state. Although we did not isolate microglia for our study, we detected a temporal-dependent changes in homeostatic gene expression in *C3*iKOmice in which there was a reduction in Tmem119, Tgfb1 and Tgfbr1 in 7-8 mo-old mice 5 mo after TAM treatment and an increase in the same genes in 16-17 mo-old mice one-year after TAM treatment compared to age-match CO treated control mice . Thus, it appears that C3 lowering may return microglia back to a homeostatic state over time.

The correlation between global C3 lowering and decreased *APOE* mRNA expression in the brain of *C3*iKO mice is intriguing, especially given that this pattern persisted for at least one-year post-TAM treatment. Germline *C3*KO mice showed the same pattern of reduced *APOE* mRNA in the brain. ApoE is heavily implicated as a risk factor in AD as well as in other diseases. In recent years, some studies have proposed that ApoE as a direct inhibitor in resolving inflammation and neuroinflammation due to its role in the blocking of a complement component C5 and attenuating the impact of the C1q-ApoE complex. Interestingly, a synergistic relationship between C3 and *APOEε4* was described in CSF in the setting of neurodegeneration. Targeting ApoE and complement might be a novel therapeutic strategy. A transcriptomic analysis in human brains carrying *APOE* 2/3 alleles identiefied complement pathway genes (C4A and C4B) as the most significantly expressed genes in 2/3 cases compared to controls. These data suggest a protective role for the complement pathway and its effects on lowering AD risk in *APOE* ε2 carriers.

In our study, we analyzed the mRNA levels of some interferon (IFN) family members (*IFNa* and *IFNb*). Type I IFN cytokines are produced in response to viral infection and are involved in the immune response and pathology of several diseases, including autoimmune diseases and cancer. We show that one year after the last injection of TAM, global C3 lowering induced a significant reduction of mRNA *IFNa* and *IFNb* levels in the brain of aged mice. These data support a previous study where the authors demonstrated that blocking IFN resulted in C3 lowering, causing a diminished microglia-mediated engulfment of synapses in an AD-like mouse model, supporting the idea that IFN can also play a pivotal role in neuroinflammation in AD.

Synapse loss has been characterized as a hallmark for several diseases. As previously discussed, complement components have been shown to increase in the CNS during aging and are known to contribute to synapse elimination mechanisms. We found a significant increase in synapses, dendritic spines and some pre-synaptic markers in the hippocampi of our *C3*iKO mice upon global C3 lowering. We and others have shown that C3 deficiency protects against synapse loss in mouse models for neurological diseases and aging. A recent report highlights the significant ability of suppressing C3a, C5a, and C5b-9 to prevent the development of neurodegeneration. As previously published by our group, germline *C3*KO protects against age-dependent hippocampal dysfunction (synapse and dendritic loss and LTP impairment) in aged mice (12 months-old). Aβ oligomers, which are proximal synaptotoxins found in human AD brains, have been identified as central molecules in inducing synapse loss in the early stages of AD. We found that C3 lowering protected against LTP impairment induced by S26C dimers, a form of toxic Aβ oligomers. Taken together, our current data support the rescue of age-related hippocampal function upon C3 lowering during adulthood and extend similar neuroprotective effects of germline *C3*KO previously reported by our group. Induction of C3 lowering in adulthood could be a useful strategy to protect synapses during aging and neurodegenerative diseases, including Alzheimer’s disease as reported by Singh *et al.* in an accompanying paper. However, knocking down C3 levels globally could leave patients unprotected against viruses and pathogens; therefore, brain-targeted therapies to reduce complement C3 would be safer and potentially more effective. To that end, Dujardin *et al.* (Vandenbroucke lab; unpublished results) investigate the effect of microglial- and astrocyte- specific C3 depletion in mouse models of inflammation and amyloidosis in an accompanying paper.

## Conclusion

In summary, our data indicate that C3 lowering after normal brain development confers neuroprotection against age-related hippocampal dysfunction in an inducible global *C3* conditional knockout mouse model. This model is useful because it allows for C3 lowering at any age or disease stage. We established a significant, sustained reduction of C3 levels in the serum, liver and brain of our *C3*iKO mice. Furthermore, we found that global C3 lowering in our mice confers protection against age-dependent cognitive decline, and hippocampal loss of synapses and dendritic spines. In addition, LTP impairment induced by Aβ-oligomers was also rescued after C3 lowering in our mice. Taken together, our data indicate that targeting C3, especially in brain, may be a beneficial therapeutic strategy to better protect against brain aging and possibly, Alzheimer’s disease.

## Figures and Tables

**Table 1:** Description of *C3*iKO and control mouse cohorts

Mouse Cohort	Age at TAM/CO Injections	Age at Euthanasia	Outcome Measures
A	4-5 mo-old	16-17 mo-old	Behavior; C3 and C1q levels in serum and brain; brain and liver *C3* mRNA (qRT-PCR); brain immune genes (qRT-PCR); gliosis; synaptic puncta analysis
B	7-8 mo-old	17-18 mo-old	Behavior; serum C3 and C1q levels; gliosis; C3, C1q and synaptic markers in synaptosomes
C	2-3 mo-old	4-5 mo-old; 7-8 mo-old	Serum C3 levels, brain immune genes (qRT-PCR) at 60- and 150-days post-TAM or CO treatment
D	2-3 mo-old	7-8 mo-old; 15-16 mo-old	LTP impairment by S26C dimers (7-8 mo of age); serum C3 levels and dendritic spine analysis (15-16 mo of age)

**Table 2.** Primers list for qRT-PCR experiments

	(5’ 3’) Forward	(5’ 3’) Reverse
Aldh1l1	ATTCCCAAGGGTGTGGTCAAC	CATCAGGGTGGTCTGAGAGTCTCT
APOE	GGGACAGGGGGAGTCCTAT	TTTGCCACTCGAGCTGATC
C1qa	CTCAGGGATGGCTGGTGGCC	CCTTTGAGACCCGGCCTCCCC
C1qb	GACTTCCGCTTTCTGAGGACA	GGGATTCCTGGCTCTCCCTT
C1qc	GGTCAGACCCGCAACTTC	GGGATTCCTGGCTCTCCCTT
C3	ACCCCTTCATTCCTTCCACCT	CTCTCCAGCCGTAGGACATT
C3aR1	TCGATGCTGACACCAATTCAA	TCCCAATAGACAAGTGAGACCAA
C4	GGTCAAGACCCGCAACTTC	CCAGGTATTTGAGTCCAGCA
C4bp	GGGAGGCTTCATAGAAACAG	ATTTCAGGGTGGTGTGACTT
CD11b	AGCCCCACACTAGCATCAA	TCCATGTCCACAGAGCAAAG
CD18	AAGGGAGTCATGGAGTGTGG	TTGTCCTTCCGACAGTTTCTC
CD55	ACTGTTGATTGGGACGATGAG	TGGTGGCTCTGGACAATGTA
Cre	CGGTCTGGCAGTAAAAACTAT	CAGGGTGTTATAAGCAATCCC
CX3cr1	ACCGGTACCTTGCCATCGT	ACACCGTGCTGCACTGTCC
Factor -H	TGATTGAAACCACCGTGAAA	AATAATGCTGGTTCCATC
Factor-1	TGGCATTTTATATCGGTGGCTGTTG	GCGCACTGCATCTCTTTCTCATAG
GAPDH	AGGTCGGTGTGAACGGATTTG	GGGGTCGTTGATGGCAACA
IFN-alpha	GCAATCCTAGACTCACTTCTGCA	TATAGTTCCTCACAGCCAGCCAGCAG
IFN-beta	TAGCACTGGCTGGCTGGAATGAG	GTTTCGGAGGTAACCTGTAAG
IL-10	GCTCTTACTGACTGGCATGAG	CGCAGCTCTAGGAGCATGTG
IL1-B	GCTTCAGGCAGGCAGTATC	AGGATGGGCTCTTCTTCAAAG
IL-6	CAGAGGATACCACTACCAACAG	TCTCATTTCCACCACGATTTCCC
mCr2	TCATGAGGGTACCTGGAGTCA	AAGAGGAATAGTTGACCGGTATTT
P2ry12	AGTGCAAGAACACTCAAG	GTGTTGACACCAGGCACA
Sall1	CGTGAGCGGCTGATGTTTGA	CTTAGTGGGGCGACTTGGTT
sCrry	GGAGGAGTCAAGCTAGAAGTTT	GTGTTGCAGCGGTAGGTAAC
Tgfb1	ATGCTAAAGAGGTCACCCGC	TGCTTCCCGAATGTCTGACG
Tgfbr1	TGCCATAACCGCACTGTCA	AATGAAAGGGCGATCTAGTGATG
Tmem119	GTGTCTAACAGGCCCCAGAA	AGCCACGTGGTATCAAGGAG
TNF-α	ATGGCCTCCCTCTCATCAGT	GTTTGCTACGACGTGGGCTA

## Data Availability

The datasets used and/or analyzed during the current study are available from the corresponding author on reasonable request.

## Abbreviations

AD: Alzheimer’s Disease
C3: Complement component 3
C3fl/fl: Chimeric floxed C3 mouse line
C3iKO: Inducible C3 knockdown (C3fl/fl; Rosa-26-Cre-ERT2+/−)
CNS: Central nervous system
CO: Corn oil
ES: Embryonic stem
LTP: Long-term potentiation
TAM: Tamoxifen
WT: Wild-type
i.p.: Intraperitoneal
qPCR: Quantitative Polymerase Chain Reaction
ELISA: Enzyme-Linked Immunosorbent Assay
SNYM: Spatial Novelty Y-Maze
NOR: Novel Object Recognition
DOR: Displaced Object Recognition
IFN1a, IFN1b: Interferon 1 alpha, beta
Tgfbr1, Tgfb1: Transforming growth factor beta receptor 1 and beta 1
P2ry12: Purinergic receptor P2Y, G-protein coupled 12
ApoE: Apolipoprotein E
C1q: Complement component 1q
GFAP: Glial fibrillary acidic protein
Iba-1: Ionized calcium-binding adapter molecule 1
Aβ: Amyloid Beta
DG: Dentate Gyrus
CA3: Cornu ammonis 3
CSF: Cerebrospinal fluid
DMSO: Dimethyl sulfoxide
TGF-β: Transforming growth factor-beta
TNF: Tumor necrosis factor
